# Both Maternal High-Fat and Post-Weaning High-Carbohydrate Diets Increase Rates of Spontaneous Hepatocellular Carcinoma in Aged-Mouse Offspring

**DOI:** 10.3390/nu16162805

**Published:** 2024-08-22

**Authors:** Daniel Holt, Laura Contu, Alice Wood, Hannah Chadwick, Ilaria Alborelli, Andrea Cacciato Insilla, Francesco Crea, Cheryl A. Hawkes

**Affiliations:** 1 Biomedical and Life Sciences, Lancaster University, Lancaster LA4 1YW, UKa.wood9@lancaster.ac.uk (A.W.); h.m.chadwick@lancaster.ac.uk (H.C.); 2 School of Psychological Sciences, Bristol University, Bristol BS8 1QU, UK; laura.contu@bristol.ac.uk; 3 Pathology, Institute of Medical Genetics and Pathology, University Hospital Basel, University of Basel, 4056 Basel, Switzerland; ilaria.alborelli@usb.ch; 4 Morphological Diagnostic and Biomolecular Characterization Area, Complex Unit of Pathological Anatomy Empoli and Prato, Usl Toscana Centro, 50122 Florence, Italy; 5 Cancer Research Group, Life, Health and Chemical Sciences, The Open University, Milton Keynes MK7 6AA, UK; francesco.crea@open.ac.uk

**Keywords:** maternal obesity, metabolic dysfunction-associated steatotic liver disease, hepatocellular carcinoma, glycolysis, fatty acid oxidation, high fat, high carbohydrate

## Abstract

Both maternal obesity and postnatal consumption of obesogenic diets contribute to the development of metabolic dysfunction-associated steatotic liver disease (MASLD) and hepatocellular carcinoma (HCC). However, there is no consensus as to whether diets that are high in fat or carbohydrates/sugars differentially influence the development of HCC. Moreover, the long-term effects of prenatal HF exposure on HCC and whether this is influenced by postnatal diet has not yet been evaluated. C57BL/6 dams were fed either a low-fat, high-carbohydrate control (C) or low-carbohydrate, high-fat (HF) diet. At weaning, male and female offspring were fed the C or HF diet, generating four diet groups: C/C, C/HF, HF/C and HF/HF. Tissues were collected at 16 months of age and livers were assessed for MASLD and HCC. Glucose regulation and pancreatic morphology were also evaluated. Liver tissues were assessed for markers of glycolysis and fatty acid metabolism and validated using a human HCC bioinformatic database. Both C/HF and HF/HF mice developed obesity, hyperinsulinemia and a greater degree of MASLD than C/C and HF/C offspring. However, despite significant liver and pancreas pathology, C/HF mice had the lowest incidence of HCC while tumour burden was highest in HF/C male offspring. The molecular profile of HCC mouse samples suggested an upregulation of the pentose phosphate pathway and a downregulation of fatty acid synthesis and oxidation, which was largely validated in the human dataset. Both pre-weaning HF diet exposure and post-weaning consumption of a high-carbohydrate diet increased the risk of developing spontaneous HCC in aged mice. However, the influence of pre-weaning HF feeding on HCC development appeared to be stronger in the context of post-weaning obesity. As rates of maternal obesity continue to rise, this has implications for the future incidence of HCC and possible dietary manipulation of offspring carbohydrate intake to counteract this risk.

## 1. Introduction

Hepatocellular carcinoma (HCC) is currently the fourth leading cause of cancer-related deaths worldwide, with a 5-year survival rate of less than 20% [1]. The majority of HCC cases are diagnosed in aged individuals [2] and at rates 3–4 times higher in men than women [3]. Chronic liver disease arising from viral infections including hepatitis B and C is a main contributor to the development of HCC [4]. In addition, in countries with high rates of obesity, metabolic dysfunction-associated steatotic liver disease (MASLD) and metabolic dysfunction-associated steatohepatitis (MASH) account for a rapid rise in the prevalence of HCC [5].

Global rates of obesity have more than doubled in the past 40 years, and around 30% of women of childbearing age are currently estimated to be obese [6]. Among the long-term conditions associated with maternal obesity, the risk of developing MASLD is higher in adult offspring born to obese mothers [7,8,9,10]. Recently, using a combination of chemotoxic exposure and maternal high-fat (HF) feeding, Sun et al. reported that the incidence of HCC was higher in young adult mice born to HF-fed dams and that tumour burden increased across multiple generations [11]. However, whether these findings are relevant in older offspring and/or in the absence of chemotoxic manipulation is unclear. Moreover, a possible interaction between pre- and postnatal HF diet on risk of HCC has not yet been investigated.

Overconsumption of saturated fats, carbohydrates and monosaccharides, including sucrose and fructose, contributes to the development of obesity, glucose intolerance and MASLD/MASH. Many preclinical models use HF and/or high-sucrose diets to study HCC in the context of obesity [12]. By contrast, caloric restriction of up to 40% has been reported to significantly reduce liver inflammation and fibrosis and decrease HCC-tumour burden, incidence and progression [13]. Moreover, an early study found that caloric restriction was most effective in slowing the growth of hepatocellular adenomas and adenocarcinomas in mice that underwent food restriction from weaning [14]. There is currently no consensus about the relative contribution of dietary sugar/carbohydrates and fat on the risk of developing HCC, as positive, neutral and negative associations have been reported between consumption sucrose or fatty acids (FAs) and the frequency of HCC [15,16,17]. Recent evidence also suggests that long-term consumption of low-fat, high-carbohydrate diets increase cholesterol deposition in the liver [18] and promote HCC [19]. In addition, several groups have reported significantly lower tumour burden in diethylnitrosamine (DEN)-treated mice fed an HF + low-sucrose diet compared to mice fed an HF + high-sucrose or high-sucrose-only diet [20,21]. This suggests that although dietary fats, carbohydrates and sugars can all induce metabolic disease and MASLD, they may contribute differentially to the development of HCC.

Under physiological conditions, metabolism of glucose and fat via glycolysis and β-oxidation of FAs, respectively, are mutually regulated by the glucose–FA cycle in the liver (Supplementary Figure S1). Many cases of MASLD-associated HCC are characterised by a downregulation of FA oxidation and concurrent stimulation of glycolysis, alongside the shunting of glycolytic metabolites towards anaerobic pathways [22]. By contrast, some β-catenin-activated HCCs display the opposite pattern of enhanced FA oxidation and reduced glycolysis [23]. Increasing evidence also suggests that HCC cells shuttle glucose into the pentose phosphate pathway (PPP) to sustain FA synthesis and maintain redox homeostasis [24]. In HCC cases associated with obesity and MASLD/MASH, there is evidence that both glucose consumption and FA synthesis are upregulated, while FA β-oxidation and acetyl-coA production are decreased [22]. This highlights both the heterogeneity of metabolic reprogramming in HCC and a need to disentangle the contribution of glycolysis and FA metabolism in obesity-related HCC. Moreover, as maternal HF feeding is associated with alterations in offspring hepatic lipid metabolism and glucose metabolism [25,26], such studies may provide additional insight into the role of maternal obesity on offspring risk of HCC.

This study investigated the impact of combinations of pre- and post-weaning HF or high-carbohydrate feeding on liver and pancreas pathology, as well as the development of HCC in 16-month-old male and female offspring. Assessment of molecular pathways related to liver glycolysis and FA β-oxidation were also evaluated. We found that the incidence of HCC was highest in lean males born to mothers fed an HF diet, despite the fact that these mice had less liver and pancreas pathology than offspring fed the HF diet post weaning. In non-cancerous liver tissues, there was an upregulation of genes associated with FA synthesis, while the molecular profile in tumours suggested an upregulation of the PPP and a downregulation of FA synthesis and oxidation.

## 2. Materials and Methods

Mouse model of pre- and postnatal feeding: Female C57BL/6 dams were randomly fed either a control (C) or high-fat (HF) diet (Special Diet Services, Witham, UK) as previously described [27,28]. The C diet (3.68 kcal/g) was composed of Atwater Fuel Energy (AFE) 10% fat, 20% protein and 70% carbohydrates. The HF diet (4.57 kcal/g) contained AFE 45% fat, 20% protein and 35% carbohydrates. Dams were fed the C or HF diets for 4 weeks before mating (at 18–27 weeks old) and kept on the diet during gestation and lactation. Stud C57BL/6 mice were fed the C diet throughout the experiment. Macro minerals, vitamins, and amino acid composition was matched between diets and detailed dietary information is provided in Supplementary Table S1. At weaning, male and female offspring were fed either the C or HF diet, generating four diet groups, C/C, C/HF, HF/C, and HF/HF, representing the pre- and post-weaning diet, respectively. Offspring were group-housed (average *n* = 4 mice/cage) by litter and maintained on the diet until sacrifice at 16 months of age. Offspring were assigned a unique numerical identifier so that the experimenters were blinded to maternal diet throughout the experiment until statistical analyses. Weekly *ad libitum* food intake was recorded for the first 6 weeks post weaning and daily food intake per mouse was calculated by dividing the total amount of food consumed by the number of mice per cage per day and values were then averaged across the *ab libitum* period. Mice underwent food restriction for 3 months at 6- and 12-months of age as part of a separate behavioural study [27]. During this time, mice were provided daily with sufficient food to maintain approximately 90% of their free-feeding weight. Food and caloric intake were also calculated as an average of values from the start and end of the restriction time period. All experiments were reviewed and approved by the Open University Animal Welfare and Ethics Review Board and the Home Office as per the UK Animal (Scientific Procedures) Act 1986 Amendment Regulations 2012 (PPL 70/8507). Experiments were carried out in accordance with ARRIVE guidelines.

Glucose (GTT) and insulin (ITT) tolerance tests: Offspring underwent GTT (*n* = 10–20/group) and ITT (*n* = 6–16/group) before sacrifice. For GTT, mice were fasted overnight, topical anaesthetic was applied to tails, and animals were injected i.p. with glucose (2 mg/g, dissolved in 0.9% sterile saline). Blood glucose concentrations were measured from the tail vein at baseline and 15, 30, 60 and 120 min post injection using an Accu-check blood glucose monitor (Roche, Welwyn Garden City, UK). In a subset of animals, blood samples were also collected at 0, 5, 15 and 30 min after glucose injection and plasma was processed for insulin concentrations using the Ultra Sensitive Mouse Insulin ELISA kit according to manufacturer’s instructions (Crystal Chem, Zaandam, The Netherlands). For ITT, mice were fasted for 3 h, injected i.p. with 0.35 U/kg recombinant human insulin, (Merck Life Science UK Limited, Gillingham, UK) and blood glucose was measured as in the GTT.

Tissue collection: Mice were given an overdose of sodium pentobarbital and perfused intracardially with 0.01 M phosphate-buffered saline (PBS). Blood was collected into heparinized tubes, spun down (2000 g for 5 min), and plasma was collected and stored at −80 °C. For frozen tissues, liver and pancreas were snap-frozen in isopentane on dry ice and stored at −80 °C until use. For fixed tissues, mice were additionally perfused with 4% paraformaldehyde (PFA), tissues were kept overnight in PFA at 4 °C, rinsed in PBS and stored in 30% sucrose. Tissues were then sliced on a cryostat (20 μm thickness), collected onto Superfrost microscope slides (Fisher Scientific, Loughborough, UK) and stored at −20 °C.

Liver histology and tumour identification: Liver sections were processed for H&E (Abcam, Cambridge, UK) and Picrosirius Red (Pioneer Research Chemicals, Essex, UK) staining. Images were captured on a Nikon Eclipse 80 Brightfield microscope (Nikon, Milton Keynes, UK). HCC was confirmed manually from H&E-stained tumours. Macrosteatosis was quantified from H & E images (3 images/animal, *n* = 5/sex/group) using Fiji software version 2.9.0 (NIH, MD, USA) by assessing the % area of liver covered by circular white spaces (circularity 0.1–0.54). Microsteatosis was determined by manually scoring images according to the total hepatic area affected (0 (<5%), 1 (5–33%), 2 (34–66%) and 3 (>66%), as per a published protocol [29]. Degree of liver fibrosis was quantified by calculating % area of liver positive for Picrosirius Red staining (3 images/animal, *n* = 5/sex/group) using Fiji software.

Plasma cholesterol: Plasma samples from non-fasted mice (*n* = 5/group) were processed in duplicate for high-density (HDL) and very-and low-density lipoproteins (v/LDL) using a Cholesterol Assay Kit, as per manufacturer’s instructions (Abcam, Cambridge, UK).

Immunohistochemistry: Liver and pancreas tissues were processed as described previously [28]. Tissues were washed in 0.01 M PBS, incubated with 3% H_2_O_2_ in methanol for 10 min and blocked with 15% goat serum. For insulin staining, sections underwent antigen retrieval by heating the slides in 10 mM sodium citrate + 0.01% Tween 20 at 80 °C in a water bath for 20 min. For CD45 and F4/80 staining, tissues were treated for 15 min with boiling 1 mM EDTA. Tissues were incubated overnight at 4 °C with anti-glucagon (1:350, Abcam, Cambridge, UK), anti-insulin (1:150, Santa Cruz Biotechnology, Santa Cruz, CA, USA), anti-CD45 (1:150, Fisher Scientific, Loughborough, UK) or anti-F4/80 (1:200, Cell Signalling Technology, Leiden, The Netherlands) diluted in 0.1% Triton X-100. The next day, sections were washed with PBS, incubated for 1 h at room temperature with biotinylated anti-rabbit or anti-mouse (1:400) and ABC kit (1:200, Vector Labs, Newark, USA) and developed using the glucose oxidase DAB method. For pancreas sections labelled with anti-CD45, sections were counterstained with Nuclear Fast Red (Merck, UK). Images were captured on a Nikon Eclipse 80 Brightfield microscope (Nikon, Normanton, UK). For pancreas images, islet number/mm^2^ tissue and islet area/mm^2^ tissue were quantified manually using Fiji (3 sections/animal, *n* = 5 mice/sex/group). For liver and pancreas sections, the % area of liver positive for CD45 was quantified using Fiji (3 sections/animal, *n* = 5 mice/group).

RT-qPCR: Frozen liver samples from normal liver samples from male offspring that did not develop HCC (*n* = 6/group) and tumours from male C/C (*n* = 6) and HF/C mice (*n* = 8) were incubated for at least 24 h in RNAlater-ICE (Fisher Scientific, Loughborough, UK), and mRNA was isolated using an RNeasy Plus Micro Kit using a TissueLyser LT (Qiagen, Manchester, UK). cDNA was synthesised using the Applied Biosystems™ High-Capacity cDNA Reverse Transcription Kit (Fisher Scientific). KiCqStart^®^ SYBR^®^ Green Primers (Merck, Gillingham, UK) were used to quantify levels of hexokinase 2 (*Hk2*), phosphofructokinase, liver type (*Pfkl*), pyruvate kinase M2 (*Pkm2*), glucose-6-phosphate dehydrogenase (*G6pdx*), acyl-CoA synthetase long-chain family member 4 (*Acsl4*), carnitine palmitoyltransferase 1A (*Cpt1a*), acyl-CoA dehydrogenase family member 11 (*Acad11*), sterol regulatory element-binding transcription factor 1c (*Srebp1c*) and b-actin (*Actb*) using the QuantiTect SYBR Green PCR Kit (Qiagen). For comparison between diet groups, C/HF was used as the reference group. For comparison between tumour and non-tumour tissues, the non-tumour values from the corresponding diet group (C/C or HF/C) were used as the reference value. Primer sequences are provided in Supplementary Table S2.

Western blot: Frozen liver samples of non-tumour liver samples from male offspring diet groups (*n* = 6/group) and tumours from male C/C (*n* = 6) and HF/C mice (*n* = 8) were sonicated in Ripa lysis buffer, centrifuged (10,000× *g* for 10 min at 4 °C), and the supernatant was stored at −80 °C until use. A total of 35–50 μg of protein was separated on 4–20% Tris-Glycine gels (Fisher Scientific, Loughborough, UK) and transferred onto nitrocellulose membranes that were blocked for 1 h with 8% non-fat milk. Blots were incubated overnight at 4 °C with anti-glucose-6-phosphate dehydrogenase (1:1500, Abcam), anti-ascl4 (1:500, Fisher Scientific UK), anti-glutathione peroxidase 4 (GPX4, 1:500, Cell Signalling Technology, Leiden, The Netherlands) or anti-hyperoxidized peroxiredoxin-3 (PRX3-SO_2/3,_ 1:500, Cambridge Biosciences, Cambridge, UK). Blots were subsequently developed with an ECL kit (Fisher Scientific UK), stripped and reprobed with anti-GAPDH (1:50,000, Merck) to ensure equal protein loading. Blots were performed in duplicate, the optic densities of the bands were quantified using Fiji, and final values were calculated by dividing the optic density of the protein of interest by the corresponding GAPDH value.

Bioinformatic analyses: mRNA levels of *Hk2*, *Pfkl*, *Pkm2*, *G6pd*, *Acsl4*, *Cpt1a*, *Acad11* and *Srebp1* in normal human liver (*n* = 50) and HCC tissues (*n* = 371) were investigated using the University of Alabama at Birmingham Cancer (UALCAN) interactive database, which is curated using The Cancer Genome Atlas, MET500 and Clinical Proteomic Tumor Analysis Consortium data portal [30]. Box and whisker plots including interquartile ranges and Welch’s *t*-tests were used to estimate differences in expression levels between normal and primary tumours [30,31]. 

Statistical analysis: All analyses were carried out using GraphPad Prizm (Boston, MA, USA). Data were checked for normality using the Shapiro–Wilk test. The ROUT test was used to determine and exclude statistical outliers. Offspring tumour incidence was calculated using Fisher’s exact test. Comparison of diet x time effects was calculated using a repeated measures two-way ANOVA, while sex x diet effects were analysed using two-way ANOVA with the Sidak post hoc test. A *p* < 0.05 was considered to be statistically significant. Data are presented as mean ± SEM unless stated otherwise.

## 3. Results

### 3.1. Postnatal HF Diet Induced Weight Gain, While Postnatal C Diet Increased HCC Incidence

**Figure 1:**
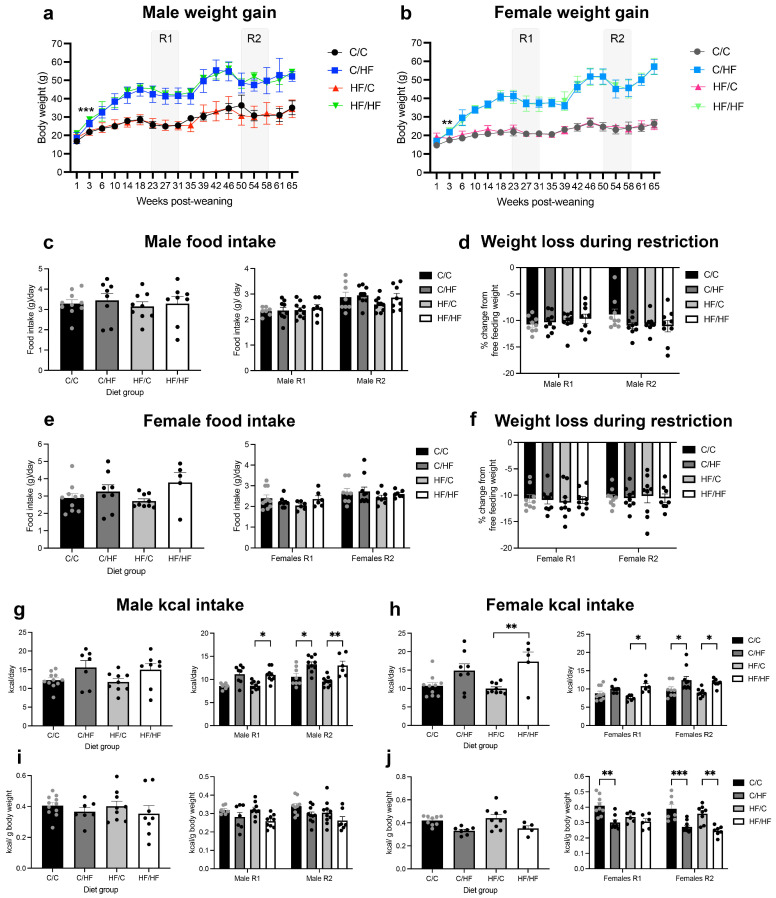
Body weight of male (**a**) and female (**b**) C/C (males = 9, females = 12), C/HF (males = 15, females = 9), HF/C (males = 9, females = 10) and HF/HF (males = 9, females = 9) offspring across their lifespan. Error bars show standard deviations. Gray areas indicate periods of food restriction (R1 and R2). ** *p* < 0.01, *** *p* < 0.001, C/HF and HF/HF mice vs. C/C and HF/C mice, two-way repeated measures ANOVA with Sidak’s post hoc. (**c**–**j**), Total food intake/day (**c**,**e**), weight loss during periods of food restriction (**d**,**f**), total kcal intake/day (**g**,**h**) and kcal/g body weight (**i**,**j**) of male (**c**,**d**,**g**,**i**) and female (**e**,**f**,**h**,**j**) offspring during *ad libitum* and restricted food intake. C/C (males = 10, females = 10), C/HF (males = 8, females = 8), HF/C (males = 9, females = 9) and HF/HF (males = 8, females = 5). * *p* < 0.05, ** *p* < 0.01, *** *p* < 0.001, two-way ANOVA with Sidak’s post hoc test. Figure adapted from [25,26].

Data regarding dam and offspring weight, as well as offspring adiposity, food and calorie consumption, have been published previously [27,28]. In brief, dams fed the HF diet weighed significantly more than *C*-fed females at mating and throughout gestation and weaning. Male and female offspring fed the HF diet postnatally (C/HF and HF/HF) also weighed significantly more than offspring fed the C diet (C/C and HF/C) from week 3 post weaning onwards (Figure 1a,b). No weight differences were observed between C/C and HF/C or between C/HF and HF/HF mice at weaning, over the life course or during periods of food restriction (Figure 1a,b). Total food intake was similar among all diet groups during periods of *ad lib* and restricted feeding (Figure 1c,e) and all offspring lost a similar amount of body weight (proportional to their free-feeding weight) during food restriction (Figure 1d,f). Male C/C, C/HF, HF/C and HF/HF mice consumed the same number of total calories during *ad lib* feeding (Figure 1g) and this pattern was maintained when adjusted by mouse body weight (Figure 1i). Intake of absolute calories was higher in HF-fed male mice than *C*-fed mice during food restriction, but not when adjusted by body weight (Figure 1g,i). HF-fed females ate more total calories than *C*-fed females during periods of both *ad lib* and restricted food intake (Figure 1h). However, when adjusted for body weight, absolute *ad lib* kcal consumption was the same between diet groups but decreased in C/HF and HF/HF females during periods of food restriction (Figure 1j).

**Figure 2:**
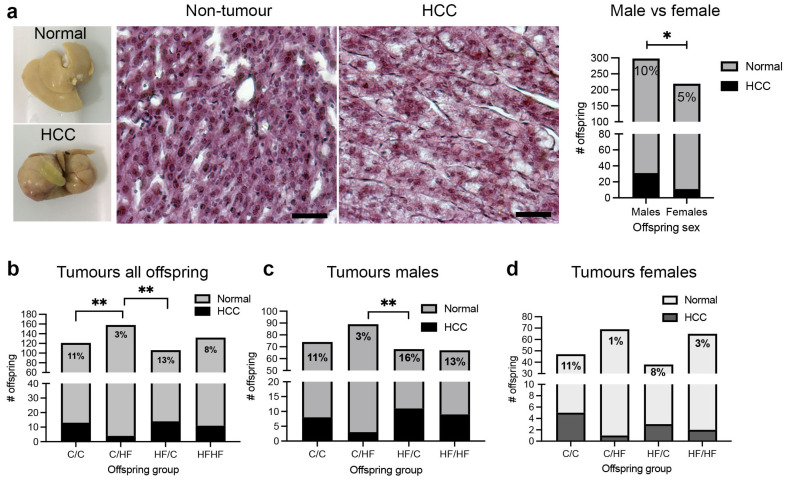
(**a**) Gross morphology of a liver without any abnormalities (normal) and with large, macroscopic tumours (HCC). H & E staining showing normal cellular architecture in non-tumour tissue and enlarged trabeculae with steatosis in an HCC tumour. Quantification of the total number of male and female offspring with and without HCC. (**b**–**d**), quantification of HCC by diet group (**b**) and within male-offspring (**c**) and female-offspring (**d**) diet groups. Inset numbers represent the percentage of animals with HCC in each group. Scale bars = 20 μm. * *p* < 0.05, ** *p* < 0.05, Fisher’s exact test.

During tissue collection from 16-month-old offspring, we observed that approximately 9% of offspring had developed macroscopic liver tumours (Figure 2a), which was more than 3-times higher than reported rates of spontaneous tumour development across the lifespan of C57BL/6 mice [32]. Pathological characterization confirmed the majority of these tumours as HCC (Figure 2a). Analysis of tumour incidence by sex revealed that tumour rate was almost twice as high in male (10%) versus female (5%) offspring (*p* = 0.03, 95% CI [0.87, 9.89]) (Figure 2a). In addition, of the combined male- and female-offspring diet groups, HF/C mice had the highest tumour incidence (13%), followed by C/C (11%), HF/HF (8%) and C/HF mice (3%) (*p* = 0.01; Figure 2b). Notably, C/HF mice had significantly lower tumour incidence than both HF/C (*p* = 0.007, 95% CI [3.78, 17.6]) and C/C offspring (*p* = 0.0045, 95% CI [2.18, 15.4]). A similar pattern of tumour incidence between diet groups was also observed in male offspring (*p* = 0.04, Figure 2c), while rates of HCC in female offspring were lower in HF/C and HF/HF mice compared to C/C, although this difference was not statistically significant (*p* = 0.10, Figure 2d).

### 3.2. Measures of MASLD Were Higher in HF-Fed Animals

**Figure 3:**
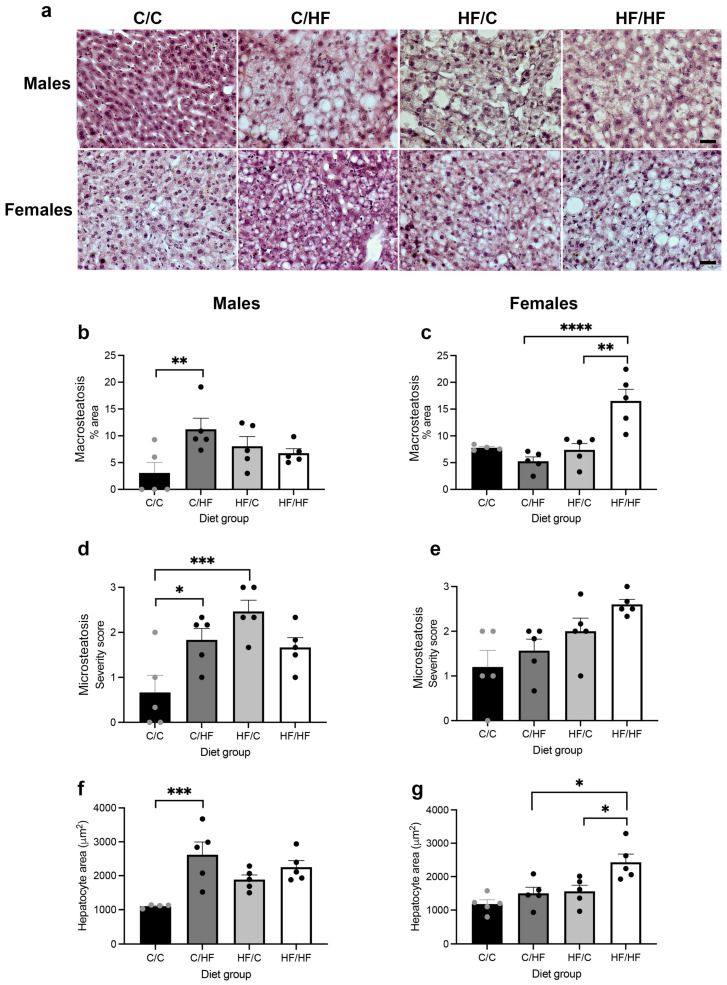
(**a**) H & E staining of liver tissues from male and female C/C, C/HF, HF/C and HF/HF offspring. Quantification of % area liver positive for macrosteatosis within male (**b**) and female (**c**) diet groups. Microsteatosis severity score between diets in males (**d**) and females (**e**). Quantification of hepatocyte area in male (**f**) and female diet groups (**g**). Scale bars = 50 μm. C/C (males = 4–5, females = 5), C/HF (males = 5, females = 5), HF/C (males = 5, females = 5) and HF/HF (males = 5, females = 5). * *p* < 0.05, ** *p* < 0.01, *** *p* < 0.001, **** *p* < 0.0001, two-way ANOVA with Sidak’s post hoc test.

To determine if the incidence of HCC was related to the effect of pre- and/or post-weaning HF diet on liver pathology, markers of liver steatosis, fibrosis and inflammation were assessed. As shown in Figure 3, macrosteatosis was significantly higher in male C/HF vs. C/C animals and in female HF/HF vs. C/HF and HF/C female offspring (Figure 3a,b). Male C/HF mice also had significantly greater macrosteatosis than female counterparts, while macrosteatosis was higher in HF/HF females than male animals (Supplementary Figure S2a). Analysis of microsteatosis found that both C/HF and HF/C male offspring had significantly greater microsteatosis compared to C/C males (Figure 3d). A similar, non-significant pattern was observed in female mice (Figure 3e), while no significant differences in degree of microsteatosis were observed between males and females (Supplementary Figure S2b). Average hepatocyte area was also significantly greater in C/HF vs. C/C males (Figure 3f) and in HF/HF females vs. C/HF and HF/C females (Figure 3g). Male C/HF mice had significantly higher hepatocyte area than females in the same diet group (Supplementary Figure S2c). 

**Figure 4:**
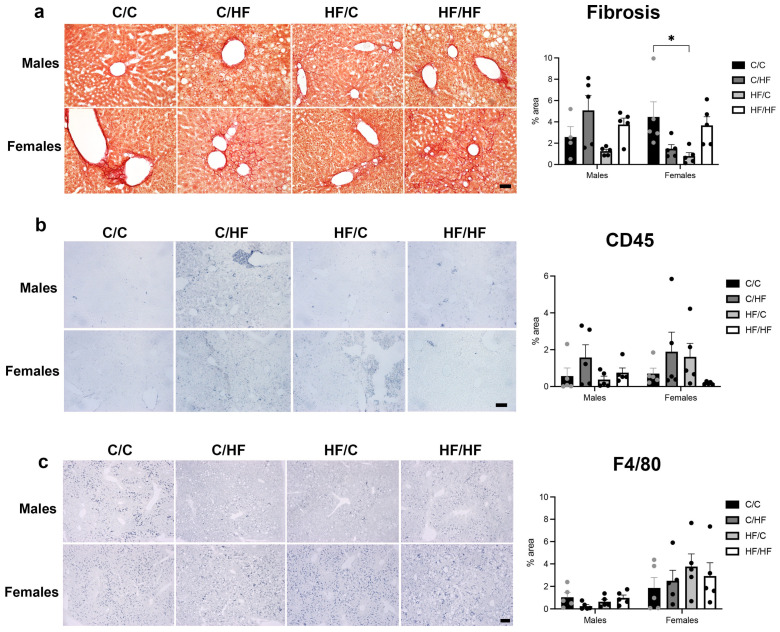
(**a**) Picrosirius Red staining of liver tissues from C/C, C/HF, HF/C and HF/HF offspring and quantification of % area liver positive for fibrosis between diet groups. (**b**) CD45 staining and quantification of liver tissues in different diet groups. (**c**) Images of F4/80-positive macrophages and quantification of % area liver positive for F4/80 staining of livers from different diet groups. Scale bar: (**a**) = 50 μm, (**b**,**c**) = 200 μm. C/C (males = 4–5, females = 5), C/HF (males = 5, females = 5), HF/C (males = 5, females = 5) and HF/HF (males = 5, females = 5). * *p* < 0.05, two-way ANOVA with Sidak’s post hoc test.

Liver fibrosis did not differ significantly between male diet groups (Figure 4a). In female mice, the HF/C group had significantly less fibrosis than C/C animals (Figure 4a) and C/HF females also showed significantly less fibrosis than male C/HF animals (Supplementary Figure S2d). Quantification of CD45 (Figure 4b and Supplementary Figure S2e) and F4/80 (Figure 4c) found no significant differences between male or female diet groups, although F4/80 staining was significantly higher in female C/HF and HF/C mice vs. males in the same diet group (Supplementary Figure S2f).

Finally, analysis of non-fasted plasma HDL concentrations found no significant differences between diet groups (Supplementary Figure S3a,b). In male offspring, HF/C mice had significantly lower v/LDL compared to C/C and HF/HF animals, while no differences were noted between female diet groups (Supplementary Figure S3c,d). In general, female mice showed lower cholesterol levels than male animals, except for v/LDL concentrations in HF/C animals, which did not differ between sexes.

### 3.3. Postnatal HF, but Not C Diet, Induced Glucose Intolerance and Hyperinsulinemia

**Figure 5:**
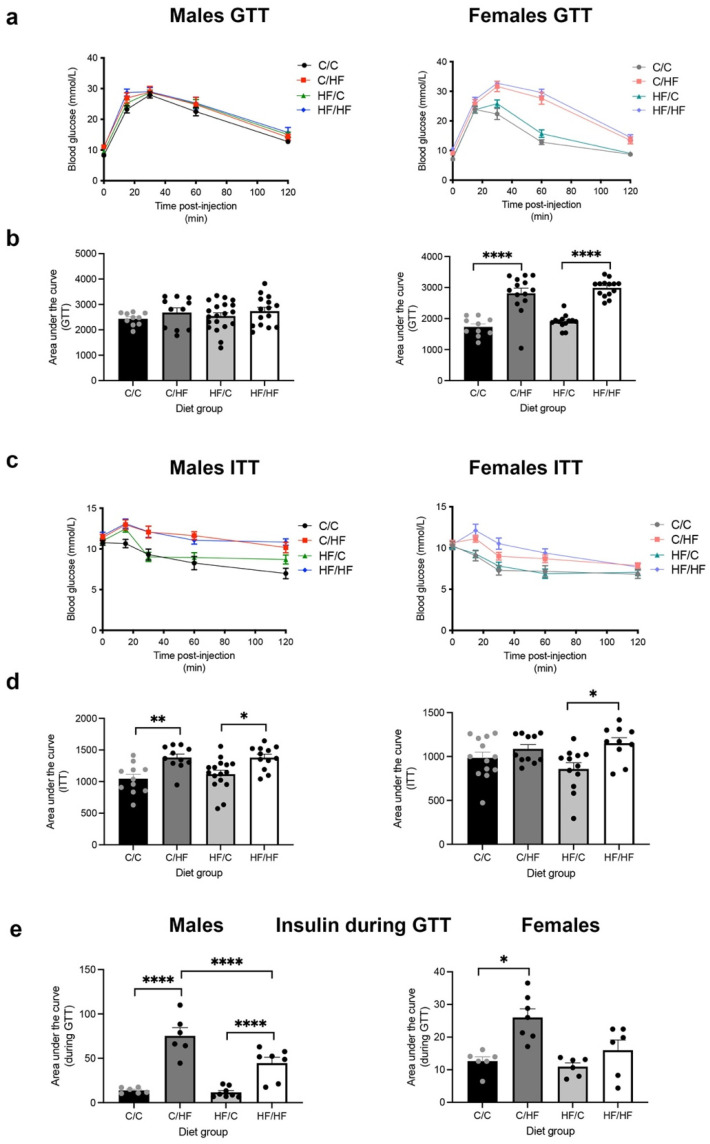
Time course (**a**) and area under the curve (**b**) of plasma glucose concentrations during the glucose tolerance test (GTT) test in 16-month-old C/C, C/HF, HF/C and HF/HF male and female offspring. C/C (males = 11, females = 10), C/HF (males = 11, females = 14), HF/C (males = 20, females = 13) and HF/HF (males = 15, females = 14). Time course (**c**) and area under the curve (**d**) during insulin tolerance test (ITT). C/C (males = 11, females = 13), C/HF (males = 11, females = 11), HF/C (males = 16, females = 12) and HF/HF (males = 12, females = 10). (**e**) Area under the curve of plasma insulin concentrations released during the GTT. C/C (males = 6, females = 6), C/HF (males = 6, females = 7), HF/C (males = 8, females = 6) and HF/HF (males = 7, females = 6). * *p* < 0.05, ** *p* < 0.01, **** *p* < 0.0001, two-way ANOVA with Sidak’s post hoc test.

Previous reports have suggested a correlation between hyperinsulinemia and increased risk of HCC [33]. Blood glucose concentrations were similar between all male-offspring diet groups during the GTT (Figure 5a,b). However, glucose levels were significantly higher in C/HF and HF/HF mice during the ITT (Figure 5c,d). Female C/HF and HF/HF mice demonstrated significantly higher blood glucose concentrations compared to C/C and HF/C animals, respectively, during the GTT (Figure 5a,b). Female HF/HF mice also had significantly higher blood glucose levels than HF/C females in the ITT. During GTT and ITT, blood glucose concentrations were significantly lower in C/C, C/HF and HF/C female mice compared to their male counterparts (Supplementary Figure S4a,b). Analysis of plasma insulin concentrations during the GTT showed that male C/HF and HF/HF mice and female C/HF animals were hyperinsulinemic (Figure 5e). Similarly, male C/HF and HF/HF mice had higher insulin levels following glucose administration compared to female offspring (Supplementary Figure S4c).

**Figure 6:**
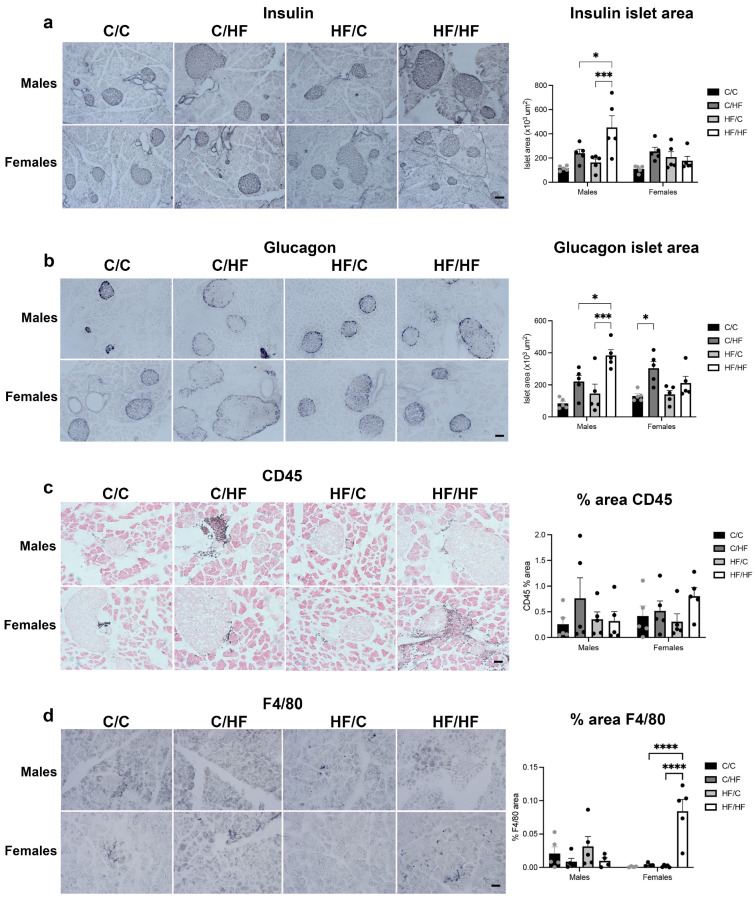
Photomicrographs and quantification of islet area in pancreases of 16-month-old male and female C/C, C/HF, HF/C and HF/HF offspring stained with insulin (**a**), glucagon (**b**), CD45 (**c**) and F4/80 (**d**). *n* = 5 for all groups. Scale bars = 100 μm. * *p* < 0.05, *** *p* < 0.001,**** *p* < 0.0001, two-way ANOVA with Sidak’s post hoc test.

In the pancreas, the average area of insulin-positive islets was significantly higher in male HF/HF mice vs. both C/HF and HF/C males (Figure 6a) and compared to HF/HF females (Supplementary Figure S4d). No differences were noted between diet groups or sex in the density of insulin-positive islets (Supplementary Figure S4e,f). A similar pattern was observed for glucagon staining, with larger glucagon-positive islets in male HF/HF mice vs. C/HF and HF/C males and in C/HF vs. C/C female offspring (Figure 6b and Supplementary Figure S4g–i). No differences in CD45 staining were seen between either male or female diet groups or between male and female offspring (Figure 6c and Supplementary Figure S4i). Similarly, F4/80 expression was unaltered between male diet groups, but significantly higher in female HF/HF mice vs. C/HF and HF/C animals (Figure 6c). Levels of F4/80 were also higher in the pancreas of female HF/C and HF/HF mice than male offspring (Supplementary Figure S4k).

### 3.4. Markers of Glycolysis and Fatty Acid Oxidation Were Altered in Both Diet Groups and in HCC

**Figure 7:**
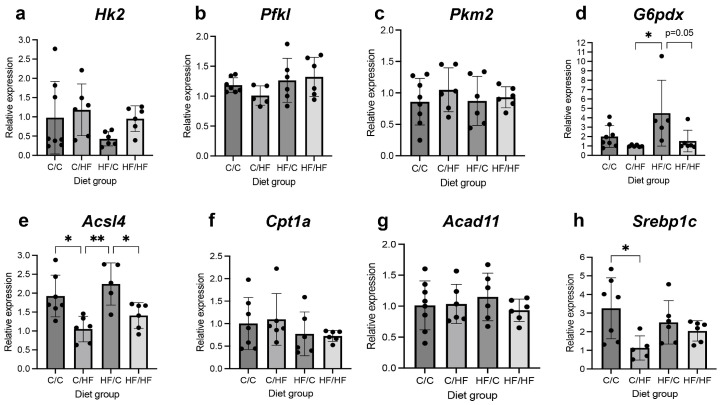
mRNA levels of *Hk2* (**a**), *Pfkl* (**b**), *Pkm2* (**c**), *G6pdx* (**d**), *Acsl4* (**e**), *Cpt1a* (**f**), *Acad11* (**g**) and *Srebp1c* (**h**) in non-cancerous liver tissues from 16-month-old male C/C (*n* = 7–8), C/HF (*n* = 6), HF/C (*n* = 6) and HF/HF (*n* = 6) offspring. * *p* < 0.05, ** *p* < 0.01, one-way ANOVA with Sidak’s post hoc test.

To determine the effect of dietary manipulation on markers of glycolysis and the PPP, mRNA levels of the rate-limiting enzymes *Hexokinase* 2 (*Hk2*), *Phosphofructokinase*, *liver type* (*Pfkl*), *Pyruvate kinase M2 (Pkm2*) and *Glucose-6-phosphate dehydrogenase* (*G6pdx*), were assessed in non-tumour livers from male offspring that did not develop HCC. mRNA levels of *Hk2* did not differ significantly between diet groups (Figure 7a). Levels of *Pfkl* and *Pkm2* were also similar between diet groups (Figure 7b,c), while *G6pdx* expression in HF/C animals was approximately 4-fold higher than in C/HF and HF/HF animals (Figure 7d). Gene expression of three enzymes involved in various steps of FA activation (*Acyl-CoA synthetase long-chain family member 4*, *Ascl4*), transport across the mitochondrial membrane (*Carnitine palmitoyltransferase 1A*, *Cpt1a*) and β-oxidation (*Acyl-CoA dehydrogenase family, member 11*, *Acad11*) was also evaluated in non-tumour tissues. mRNA levels of *Ascl4* were significantly upregulated in C/C and HF/C offspring compared to C/HF animals (Figure 7e), while expression of *Cpt1a* and *Acad11* was similar between diet groups (Figure 7f,g). Given that ACSL4 can regulate the expression of Sterol Regulatory Element-Binding Protein 1c (SREBP-1c), a master regulator of genes required for FA synthesis [34], *Srebp1c* expression was also evaluated and found to be significantly higher in C/C mice compared to C/HF animals (Figure 7h).

**Figure 8:**
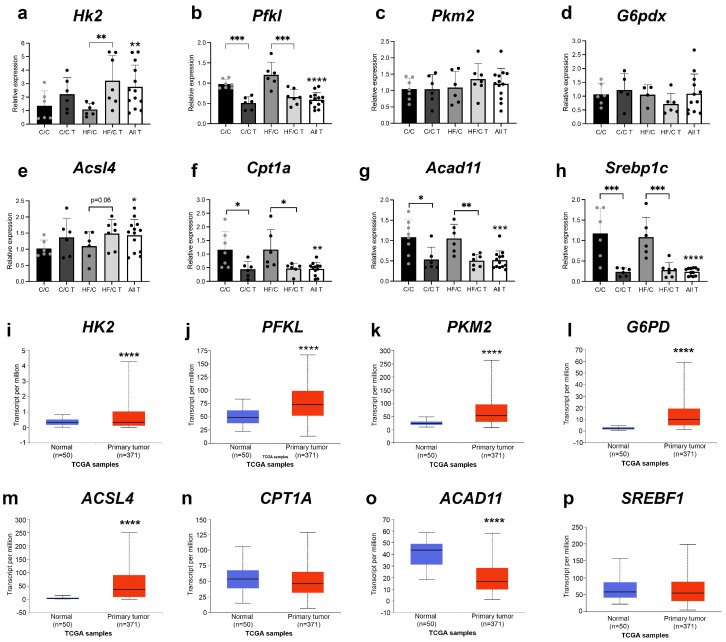
mRNA levels of *Hk2* (**a**), *Pfkl* (**b**), *Pkm2* (**c**) and *G6pdx* (**d**), *Acsl4* (**e**), *Cpt1a* (**f**), *Acad11* (**g**) and *Srebp1c* (**h**) in non-cancerous liver and tumours (T) from male C/C and HF/C mice. C/C (normal = 7, HCC = 6), HF/C (normal = 6, HCC = 7). The relative expression of genes in pooled tumours from both C/C and HF/C mice is represented in the all-tumours (All T) group. * *p* < 0.05, ** *p* < 0.01, *** *p* < 0.001, **** *p* < 0.0001, two-tailed Student’s t-test. mRNA levels of *HK2* (**i**), *PFKL* (**j**), *PKM2* (**k**), *G6PD* (**l**), *ACSL4* (**m**), *CPT1A* (**n**), *ACAD11* (**o**) and *SREBF1* (**p**) in normal human liver (blue) and HCC (red) tissues. Box and whisker plots were downloaded from the UALCAN website. **** *p* < 0.0001, Welch’s *t*-tests.

To determine if patterns of gene expression were similar or opposite in HCC tissues, markers of glycolysis and FA oxidation were also determined in tissues from tumours collected from male C/C and HF/C mice and compared to normal liver tissues from C/C and HF/C mice that did not develop HCC. Gene expression from biometric data obtained from human HCC samples in the UALCAN database was also evaluated. In mouse tis-sues, *Hk2* expression was significantly increased in tumours from HF/C mice, while *Pfkl* expression was significantly decreased in tumours from both C/C and HF/C mice (Figure 8a,b). *Pkm2* and *G6pdx* levels were unchanged relative to levels in the corresponding non-tumour diet group (Figure 8c,d). mRNA levels of *Ascl4* were not significantly different between groups, although there was a pattern of increased expression in tumours from C/C and HF/C animals (Figure 8e), while levels of *Cpt1a*, *Acad11* and *Srebp1c* were all significantly decreased in C/C and HF/C tumours relative to non-tumour tissues (Figure 8f–h). In human HCC samples, mRNA levels of *HK2*, *PFKL*, *PKM2* and *G6PD* were all significantly increased in HCC compared to normal liver tissues (Figure 8i–l). *ACSL4* expression was also significantly higher in HCC (Figure 8m), while *CPT1A* levels were unaltered (Figure 8n) and *ACAD11* was significantly decreased in HCC tissues (Figure 8o). mRNA levels of the human sterol regulatory element-binding transcription factor 1 (*SREBF1*) gene were also unchanged between normal and HCC samples (Figure 8p).

**Figure 9:**
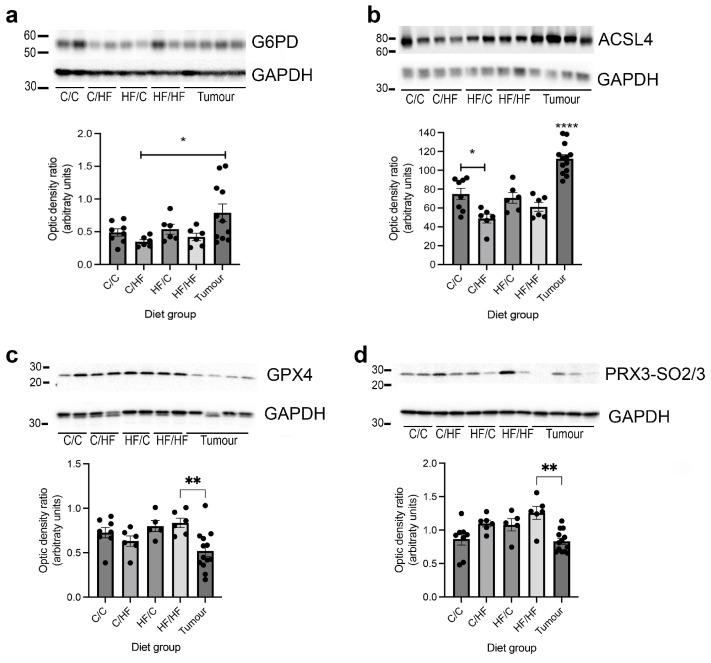
Western blots of G6PD (**a**), ASCL4 (**b**), GPX4 (**c**) and PRX3-SO_2/3_ (**d**) expression in non-cancerous and HCC liver tissues from male mice. C/C = 7, C/HF = 6, HF/C = 5, HF/HF = 6; tumour = 13. * *p* < 0.05, ** *p* < 0.01, one-way ANOVA with Sidak’s post hoc test. For panel (**b**), **** *p* < 0.0001 represents the fact that ASCL4 expression in the tumour samples is significantly different from all other groups.

Finally, to confirm if protein levels of genes that were differentially expressed between mouse diet groups and/or tumours were similarly altered, Western blots of mouse non-tumour and tumour tissues were analysed for expression of G6PD, ASCL4 and SREBP1. G6PD levels were significantly increased in HCC tissues vs. C/HF mice (Figure 9a). ASCL4 protein levels were significantly higher in C/C vs. C/HF mice and in tumours, compared to normal tissues in all other diet groups (Figure 9b). SREBP1 proteins were not detected in cytosolic liver fractions. In addition, because of the inter-relationship between the glycolysis-FA cycle and ferroptosis (Supplementary Figure S1), an iron-dependent mechanism of cell death that is thought to exacerbate liver inflammation and fibrosis preceding HCC development [35], expression of the GPX4 liver enzyme and the ferroptosis marker PRX3-SO_2/3_ [36], was also analysed. Neither GPX4 nor PRX3-SO_2/3_ was significantly different between diet groups in normal liver tissues, although both were significantly lower in HCC samples (Figure 9c,d).

## 4. Discussion

Both maternal obesity and postnatal consumption of high-fat or high-carbohydrate diets are associated with increased risk of MASLD/MASH, but their combined effects on the risk of spontaneous HCC have not yet been evaluated. In the current study, post-weaning HF feeding resulted in higher body weight, hyperinsulinemia and related pancreatic pathology, and a greater degree of MASLD than *C*-fed offspring. Despite greater liver pathology in HF-fed mice, the incidence of HCC was lowest in C/HF males and highest in HF/C animals. The molecular profile of tumours that developed in C/C and HF/C offspring suggested an upregulation of the PPP and a downregulation of FA synthesis and oxidation.

Numerous studies suggest a role for developmental priming of MASLD and MASH in offspring exposed to an HF diet during gestation and/or lactation [7,8,9,37]. A positive association between maternal pre-pregnant BMI and risk of MASLD in children has also been reported clinically [38]. Maternal HF feeding has recently been directly implicated in the development of spontaneous HCC [39] and following chemotoxic pre-treatment in mouse offspring [11]. Thus, the current observation of increased microsteatosis and development of HCC in 16-month-old HF/C offspring is consistent with previous reports. Interestingly, the influence of maternal HF feeding on HCC development appeared to be stronger in the context of post-weaning obesity, as overall rates of HCC were similar between C/C and HF/C mice but ~3-fold higher in HF/HF vs. C/HF offspring. Relative to C/HF mice, HF/HF animals had a greater degree of pancreas-islet hypertrophy and lower levels of hyperinsulinemia, suggesting that HF/HF mice had more advanced glucose intolerance, which may have increased the risk of HCC [40]. However, additional work is needed to determine the mechanisms underlying the influence of maternal diet on HCC risk in the context of a lean and obese postnatal environment.

Rates of HCC were 2x higher in male HF/C vs. HF/C females, similar to the sex imbalance observed in human HCC [3]. This is also congruent with findings that male offspring of HF-fed mothers develop greater liver steatosis than female mice [41] and with our observations that, across all diet groups, female offspring had less liver and pancreas pathology and delayed onset of glucose intolerance compared to their male littermates. Intriguingly, in *C*-diet-fed mice, the development of HCC occurred in the absence of altered glucose metabolism or significant liver fibrosis and inflammation. Experimental evidence suggests that the relative ratio of dietary fat and sucrose are important in the initiation and propagation of HCC. For example, Healy et al. found that tumour burden and size was highest in DEN-treated mice fed a 23% fat + 21% sucrose or 31% sucrose + 15% fructose diet, while those fed a 71% fat + low-sucrose diet had the lowest tumour incidence [20,21]. Duan et al. [42] reported that DEN-treated rats fed with a diet high in saturated FAs developed fewer and smaller HCC tumours than *C*-fed animals. Moreover, there is some evidence that consumption of ketogenic diets where the fat:carbohydrate ratio is 4:1 or 3:1 can delay tumour growth and prolong survival time, although clinical trials in HCC are limited [43]. In the current study, the C diet had lower fat composition, but higher carbohydrate and sucrose content compared to the HF diet (Supplementary Table S1). As sustained intake of high-carbohydrate diets is associated with increased hepatic glucose [44], and has been associated with development of HCC in aged mice [19], this may account in part for the increased HCC observed in C/C and HF/C animals. In addition, elevated consumption of fructose, which is an intermediary in glucose metabolism, is associated with the development of MASLD and increased risk of HCC [45,46]. Moreover, fructose is one of the primary metabolites used for *de novo* lipogenesis, including synthesis of triglycerides (TG) [45]. Inhibition of TG secretion packaged as vLDL is associated with steatosis in the absence of hepatic inflammation or insulin resistance [47]. Thus, the observed decrease in plasma v/LDL in HF/C males suggests that either impaired vLDL secretion and/or TG accumulation may have contributed to HCC development in these animals. However, these results must be interpreted with caution because the effect of the post-prandial interval on cholesterol concentrations was not controlled for in the non-fasted mice. Whether this is sufficient to drive HCC in the absence of other liver pathologies requires further investigation, although, notably, low-serum-LDL concentrations are independent predictors of HCC in humans [48].

In offspring fed the HF diet, there was a negative association between liver and pancreas pathology and prevalence of HCC. This was unexpected, considering the relationship between MASLD/MASH, glucose intolerance and increased risk of HCC [49]. A recent study by Pedersen et al. found that HCC incidence was higher in DEN-treated mice fed a diet enriched with saturated FAs compared to those containing monosaturated FAs (MUFAs) and polyunsaturated FAs (PUFAs) [50]. Here, 19% of the fat in the C diet was derived from saturated FAs, while MUFAs and PUFAs contributed 27% and 54%, respectively, of the fat content. In the HF diet, saturated FAs and MUFAs both made up 38% of the fat content, while the remaining 24% was contributed from PUFAs. This differential FA composition is interesting, in light of the relative contribution of MUFAs and PUFAs to hepatic ferroptosis. In ferroptosis, PUFAs that are produced via the activity of ASCL4 undergo peroxidation, leading to the generation of reactive oxygen species and cell death [51]. However, the ability of PUFAs to stimulate ferroptosis is competitively blocked by exogenous MUFAs [52] and by the actions of GPX4, which converts lipid peroxidases back to their respective alcohols using NADP+ that is generated in the PPP [51]. GPX4 activity is directly inhibited by ASCL4 [51]. In HCC itself, hepatocytes upregulate protective mechanisms to counteract ferroptosis and evade death [53]. Therefore, the relatively high concentrations of MUFAs in the HF diet, in conjunction with elevated concentrations of saturated FAs that are converted to MUFAs in the liver [54], may have inhibited ferroptosis and delayed or counteracted the onset of HCC in the C/HF mice. However, while levels of GPX4 and the ferroptosis marker PRX3-SO_2/3_ were downregulated in mouse HCC samples, neither protein was altered in livers from non-cancerous C/HF mice. It may be that ferroptosis did not play a significant role in the induction of HCC in this mouse model or that the liver samples in the animals that did not develop HCC were resistant to this pathway. More work is needed to determine why most C/HF mice did not develop HCC despite having a high degree of MASLD.

Although changes to both glycolysis and FA metabolism have been implicated in HCC, there is significant heterogeneity in the metabolic reprogramming of each pathway, depending on the metabolic background in which the HCC develops. In the current study, *Gpdx* mRNA levels were significantly increased in non-cancerous liver tissues of HF/C mice, relative to C/HF and HF/HF animals. No differences were observed between diet groups on other markers of glycolysis. However, in mouse HCC samples, levels of *Hk2* mRNA and G6PD protein were increased. These changes match those observed in the human HCC samples. This suggests that the HCC that developed in C/C and HF/C animals may have been related to increased glucose shunting to the PPP, which is consistent with reports that the PPP is a key pathway by which HCC cells sustain FA synthesis and maintain redox homeostasis [24].

In the FA pathway, expression of *Acsl4* and *Srebp1c* was higher in normal tissues from diet groups that were more likely to develop HCC. This is consistent with previous work showing that a high-carbohydrate, low-fat diet induces FA synthesis in hepatocytes [55]. Notably, *Acsl4* mRNA and protein levels were also significantly upregulated in HCC tissues, suggesting that this may be an early alteration in preneoplastic lesions. However, in contrast with non-tumour tissues, mRNA levels of *Cpt1a*, *Acad11* and *Srebp1c* were downregulated in mouse HCC. Again, these results mirror the changes in *ACSL4* and *ACAD11* expression in human HCC tissues. Although decreased FA β-oxidation has been previously reported in HCC associated with obesity [22], most studies report a positive association between SREBP1c expression and increased risk of HCC [56]. Additional experiments are needed to determine the impact of *Srebp1c* downregulation and to disentangle the seemingly paradoxical relationship between elevated levels of ACSL4 and poor HCC prognosis [57], alongside the role for ACSL4 in PUFA activation and increased sensitization to ferroptosis [58].

Although this is the first study to characterize the combined effect of pre- and post-weaning diet on rates of HCC in aged animals, it does have several limitations. Due to the longitudinal nature of the study, pathological examination of HCC could only be carried out at one time point across the offspring lifespan. Consequently, it is not clear when the HCC began or if the non-cancerous tissues would have become cancerous with more advanced aged. In addition, all mice underwent two periods of food restriction (at 6 and 12 months of age), during which the animals were maintained at 90% of their free-feeding body weight [28]. A recent systematic review of animal models of hepatic cancer concluded that caloric restriction of 20–30% delayed tumour development and reduced tumour burden and metastasis [13]. In this study, all male offspring consumed the same amount of total food/day and kcal/body weight across their lifespan. Nevertheless, we cannot discount the fact that this restriction and re-feeding paradigm may have differentially affected the metabolism and relative risk of developing HCC in lean vs. obese animals. An early study by Gumaa and Mclean [59] found that rats fasted for 72 h had decreased concentrations of liver pentose phosphate and that levels were restored in animals re-fed a high-carbohydrate diet but not animals who were re-fed with an HF diet. Whether a similar effect is observed in food-restricted mice and in the context of obesity remains unknown. Additional experiments without food restriction are needed to confirm the role of pre- and post-weaning carbohydrate and HF diets on the risk of developing spontaneous HCC. Finally, due to insufficient numbers of tumours in HF-fed mice, only HCC samples from C/C and HF/C mice were analysed. Future comparative analysis of FA β-oxidation and glycolysis in tumours that arose in lean vs. obese mice may provide additional information about the pre- and postnatal factors that contribute to HCC heterogeneity.

## 5. Conclusions

In summary, this study found that both maternal HF and post-weaning C diet increased the risk of developing spontaneous, age-related HCC in the absence of chemotoxic exposure or genetic predisposition. This may have been due to early alterations in genes related to the PPP and FA synthesis and/or oxidation, which were exacerbated by prolonged intake of a high-carbohydrate diet and attenuated by a high dietary ratio of MUFAs to PUFAs. As rates of maternal obesity continue to rise globally, this has possible implications for the future incidence of HCC, and suggests that reducing carbohydrate intake in offspring may successfully counteract this risk.

## Data Availability

The datasets used and/or analysed during the current study are available from the corresponding author on reasonable request. Human HCC bioinformatics datasets used in this study are publicly available and can be found at https://ualcan.path.uab.edu/index.html (accessed on 5 July 2024). Further enquiries can be directed to the corresponding author.

## Supplementary Materials

The following supporting information can be downloaded at: https://www.mdpi.com/article/10.3390/nu16162805/s1, Figure S1: Simplified schematic of the inter-relationship between glycolysis, the pentose phosphate pathway and fatty acid β-oxidation in hepatocytes; Figure S2: Assessment of liver steatosis and inflammation between male and female offspring; Figure S3: Plasma cholesterol concentrations; Figure S4: Measures of plasma glucose and insulin concentrations and pancreatic pathology in offspring; Table S1: Diet Composition; Table S2: Primer sequences used for RT-qPCR.
