# Supplementary material for: Both Maternal High-Fat and Post-Weaning High-Carbohydrate Diets Increase Rates of Spontaneous Hepatocellular Carcinoma in Aged-Mouse Offspring
Source: Nutrients. 2024 Aug 22;16(16):2805. doi: 10.3390/nu16162805 (PMC11357072; doi:10.3390/nu16162805)
Supplement: Supplementary file 1 [file nutrients-16-02805-s001.zip › nutrients-3166483-supplementary.pdf]

Supplemental Figures

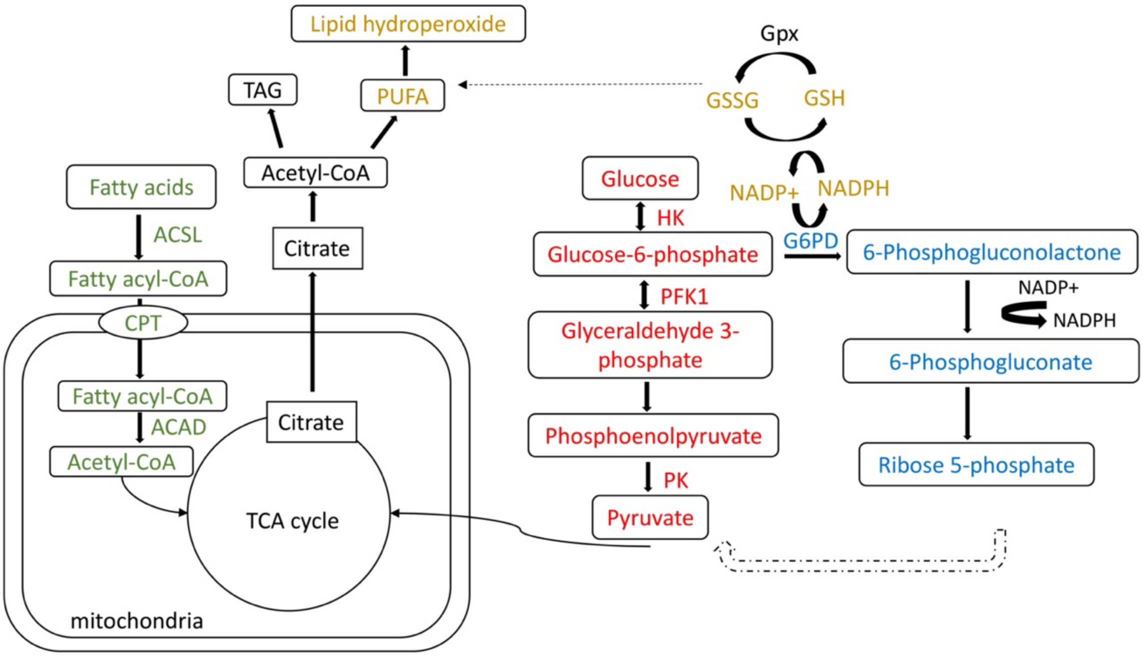

Supplemental Figure S1. Simplified schematic of the inter-relationship between glycolysis (red), the pentose phosphate pathway (blue) and fatty acid β-oxidation (green) in hepatocytes. Fatty acid metabolism and glycolysis also contribute to the regulation of ferroptosis (yellow). Dotted lines indicate indirect pathways. ACAD, acyl-CoA dehydrogenase family member; ACSL4, acyl-CoA synthetase long chain family member 4; CPT, carnitine palmitoyltransferase; G6PD, glucose-6-phosphate dehydrogenase; GPX4, Glutathione Peroxidase 4; GSH, glutathione; GSSG, oxidized glutathione; HK, hexokinase; PFKL, phosphofructokinase, liver type; PK, pyruvate kinase; PUFA, polyunsaturated fatty acids; TAG, triglycerides.

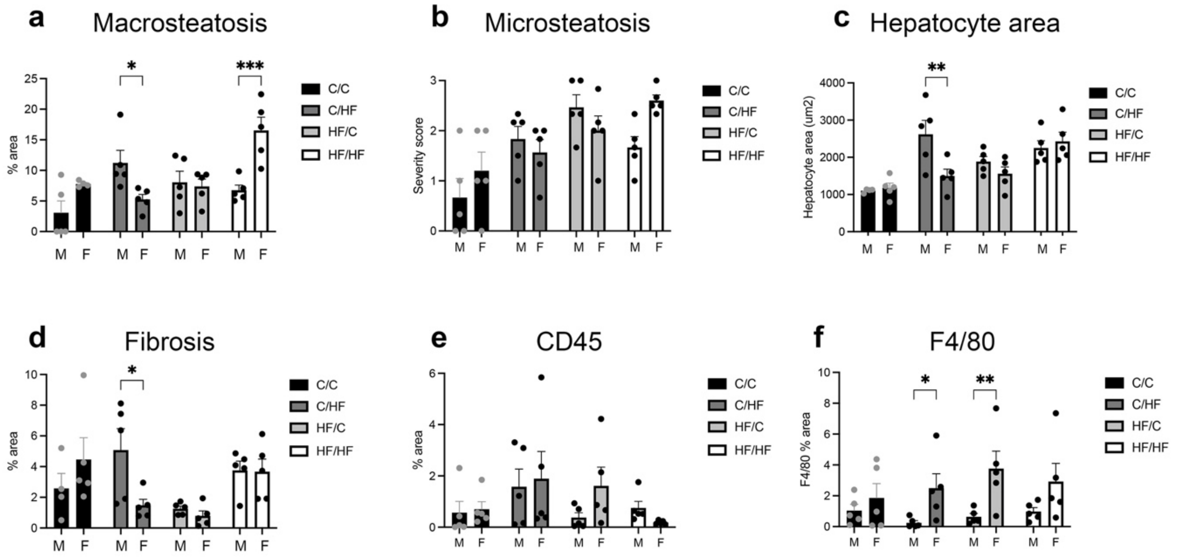

Supplemental Figure S2. Quantification of % area liver positive for macrosteatosis (a), microsteatosis severity score (b), hepatocyte area (c), fibrosis (d), and % liver area positive for CD45 (e) and F4/80 (f) between 16-month-old male and female offspring. C/C (males = 4-5, females = 5), C/HF (males = 5, females = 5), HF/C (males = 5, females = 5) and HF/HF (males = 5, females = 5). *p<0.05, **p<0.01, two-way ANOVA with Sidak's post hoc test.

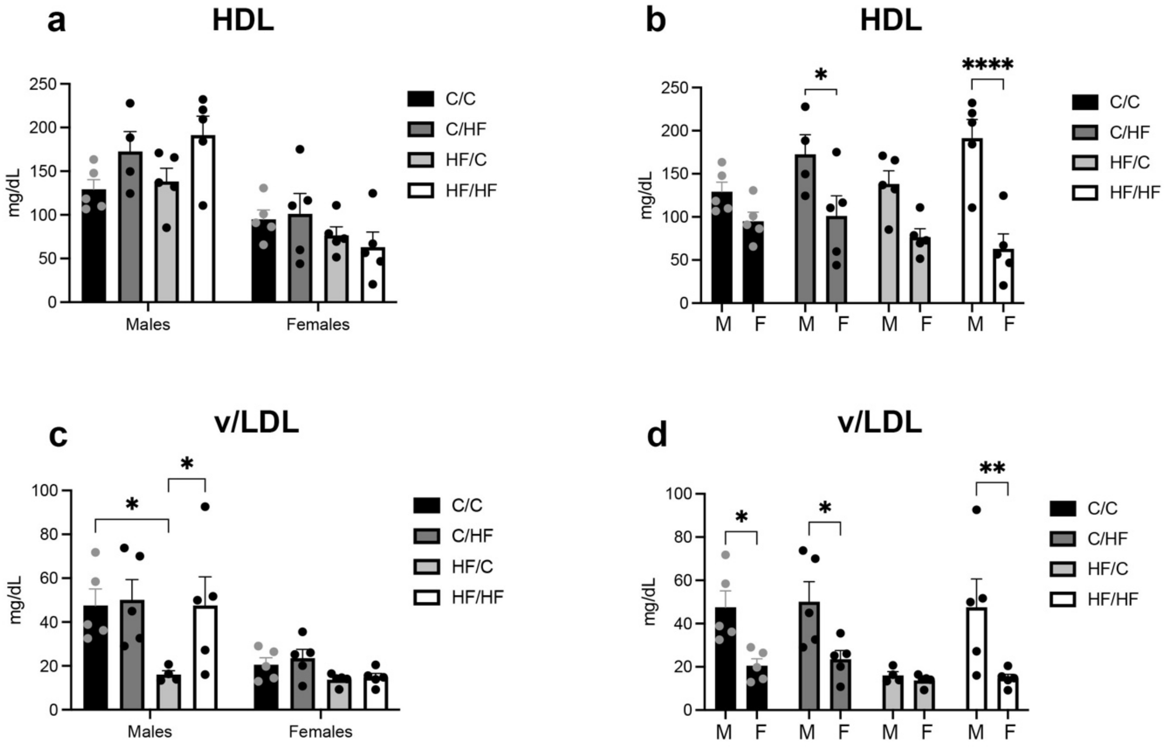

Supplemental Figure S3. Non-fasted plasma high density lipoprotein (HDL) (a and b) and very-low- and low-density cholesterol (v/LDL) (c and d) concentrations in 16-month-old male and female C/C, C/HF, HF/C and HF/HF offspring. *p<0.05, **p<0.01, ****p<0.0001, two-way ANOVA with Sidak's posthoc test.

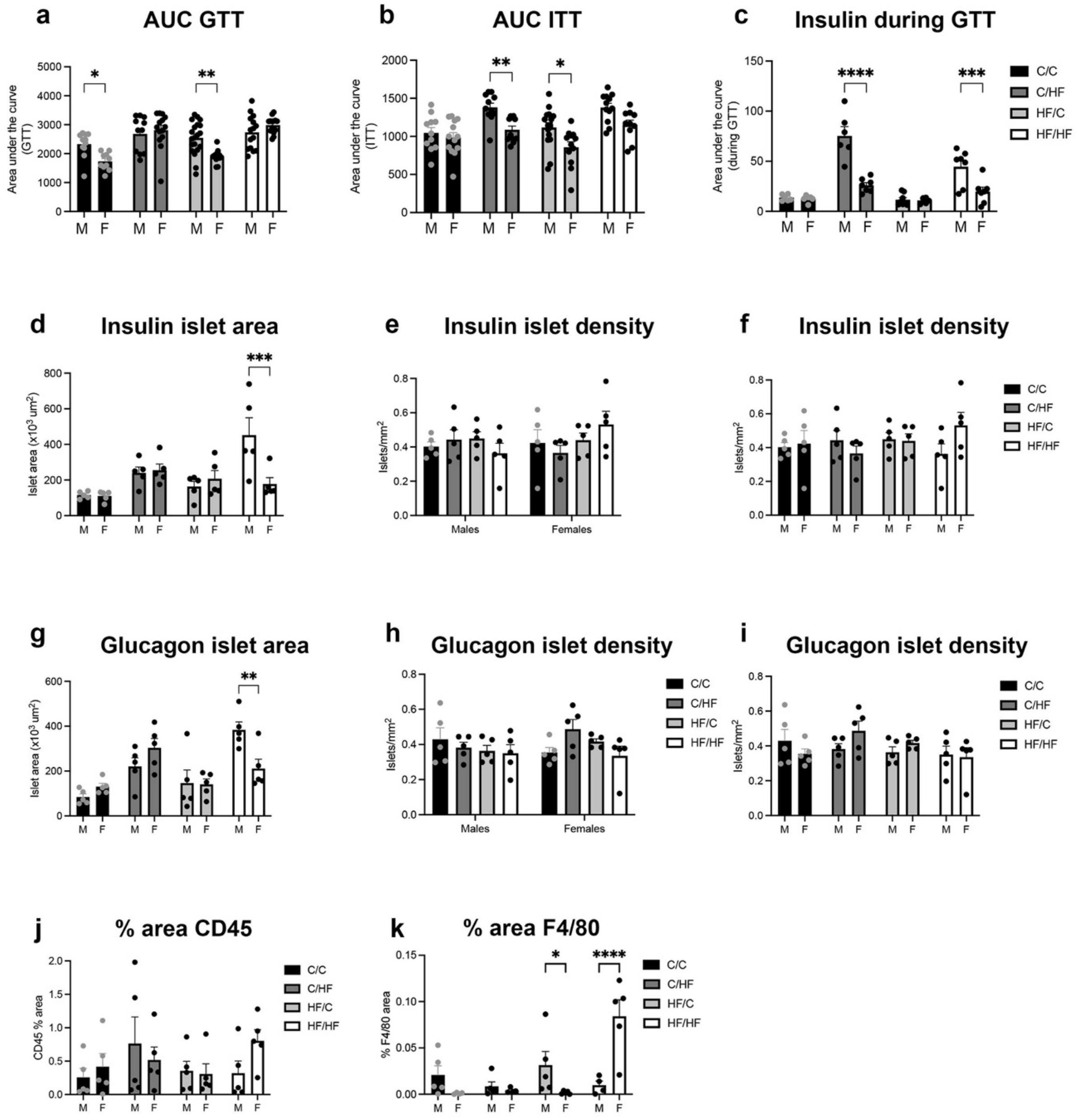

Supplemental Figure S4. Area under the curve of plasma glucose concentrations during the glucose tolerance test (GTT) test (a), the insulin tolerance test (ITT) (b) and insulin release during the GTT (c) in 16-month-old male and female offspring. C/C (males = 11, females = 10), C/HF (males = 11, females = 14), HF/C (males = 20, females = 13) and HF/HF (males = 15, females = 14). Quantification of insulin-positive islet area (d) and density (e and f) in offspring pancreases. Quantification of pancreatic staining of glucagon-positive islet area (g) and density (h and i). Comparison of the % pancreatic area positive for CD45 (j) and F4/80 (k) staining between male and female offspring. n=5 for all groups. *p<0.05, **p<0.01, ***p<0.001, ****p<0.0001, two-way ANOVA with Sidak's post hoc test.

Supplemental Table S1   Diet Composition

	Control diet	High fat diet
**Ingredients** (% inclusion)		
Casein	21.5	26.5
Choline bitartrate	0.24	0.296
L-Cystine	0.32	0.398
Lard	0	18
Rice Starch	32.44	18.43
Cellulose	5	6.164
Soya oil	3.5	4.315
Sucrose	32.5	20.34
Mineral mix	3.5	4.315
Vitamin mix	1	1.233
**Proximates** (%)		
Crude oil	3.73	21.22
Crude protein	17.59	21.55
Crude fibre	3.59	4.22
Ash	3.38	4.12
Nitrogen free extract	60.43	37.90
**Fatty acids** (%)		
C12:0 (lauric acid)	0.07	0.03
C14:0 (myristic acid)	0.12	0.29
C16:0 (palmitic acid)	0.22	4.04
C18:0 (steric acid)	0.13	1.8
C14:1 (myristoleic acid)	0.01	0.02
C16:1 (palmitoleic acid)	0.07	0.03
C18:1 (oleic acid)	0.66	6.06
C18:2 (linoleic acid)	1.26	3.53
C18:3 (linolenic acid)	0.22	0.37
C20:4 (arichidonic acid)	0.01	0.01
C22:5 (clupanodonic acid)	0	0
**Amino acids** (%)		
Arginine	0.55	0.69
Lysine (6)	1.11	1.37
Methionine	0.43	0.53
Cysteine	0.35	0.42
Tryptophan	0.16	0.19
Histidine	0.4	0.49
Threonine	0.61	0.75
Isoleucine	0.88	1.09
Leucine	1.33	1.64
Phenylalanine	0.73	0.9
Valine	1.06	1.3
Tyrosine	0.73	0.9
Taurine	0	0
Glycine	0.7	0.85

Aspartic acid	0.99	1.22
Glutamic acid	2.87	3.53
Proline	1.2	1.47
Serine	0.66	0.82
Hydroxyproline		0
Hydroxylsine		0
Alanine	0.56	0.69
**Macro minerals**		
Calcium (%)	0.46	0.59
Total Phosphorus (%)	0.18	0.35
Phytate Phosphorus (%)		0
Available Phosphorus (%)		0.35
Sodium (%)	0.12	0.15
Chloride (%)	0.22	0.26
Potassium (%)	0.46	0.42
Magnesium (%)	0.06	0.08
**Micro minerals**		
Iron (mg/kg)	44.99	55.7
Copper (mg/kg)	6.67	8.22
Manganese (mg/kg)	10.13	12.22
Zinc (mg/kg)	54.63	64.67
Cobalt (μg/kg)	0	0
Iodine (μg/kg)	194.04	238.95
Selenium (μg/kg)	138.13	185.09
Fluorine (mg/kg)		1.16
**Vitamins**		
Vitamin A (iu/kg)	3758.61	4628.65
Vitamin D3 (iu/kg)	2151.81	2822.23
Vitamin E (iu/kg)	74.09	94.46
Vitamin B1 (mg/kg)	5.61	5.73
Vitamin B2 (mg/kg)	4.86	5.98
Vitamin B6 (mg/kg)	6.53	6.7
Vitamin B12 (μg/kg)	23.49	28.93
Vitamin C (mg/kg)	0	0
Vitamin K (mg/kg)	0.68	0.89
Folic acid (mg/kg)	1.88	2.22
Nicotinic acid (mg/kg)	27.91	34.37
Pantothetic acid (mg/kg)	13.74	17.33
Choline (mg/kg)	926.94	1248.4
Inositol (mg/kg)	0	0
Biotin (μg/kg)	187.93	231.43

Supplemental Table S2 Primer sequences used for RT-qPCR

Gene	Forward primer sequence	Reverse primer sequence
Actin (*Actb*)	GATGTATGAAGGCTTTGGTC	TGTGCACTTTTATTGGTCTC
Acyl-CoA Dehydrogenase Family Member 11 (*Acad11*)	GTCAAAAAGAACTCTCAGGAC	CCACTTTGAGCATAGTATTCAG
Acyl-CoA Synthetase Long Chain Family Member 4 (*Acsl4*)	GTTCCGGAAATCATGGATAG	CAGTATCAGATTACAAAGAGGG
Carnitine palmitoyltransferase 1A (*Cpt1a*)	GGGAGGAATACATCTACCTG	GAAGACGAATAGGTTTGAGTTC
Glucose-6-phosphate 1-dehydrogenase X (*G6pdx*)	GATCATCAGGGATGTTATGC	CTCTGAGATACACTTCN1CAC
Hexokinase 2 (*Hk2*)	CAAGCTACAGATCAAAGAGAAG	CATGAGACCAAGAAACTCTC
Phosphofructokinase, Liver type (*Pfkl*)	AAGAGACTGATTTTGAGCAC	CTCAGAAACCCTTGTCTATG
Pyruvate kinase M2 (*Pkm2*)	CAGGTTTGATGAGATCTTGG	CTTCTTGATCATGCTCTCC
Sterol regulatory element-binding protein 1 (*Srebp1c*)	GGAGCCATGGATTGCACATT	CAGGAAGGCTTCCAGAGAGG
